# Associations between sodium intake and gut microbiota composition

**DOI:** 10.3389/fnut.2026.1889723

**Published:** 2026-09-07

**Authors:** Jonas Wuopio, Tiscar Graells, Yi-Ting Lin, Tessa Schillemans, Janne Marie Moll, Aron C. Eklund, H. Bjørn Nielsen, Anders Larsson, Gunnar Engström, Marju Orho-Melander, Linda S. Johnson, Johan Ärnlöv

**Affiliations:** 1 Department of Neurobiology, Care Sciences and Society, Karolinska Institute, Huddinge, Sweden; 2 Center for Clinical Research, Falun, Region Dalarna, Uppsala University, Uppsala, Sweden; 3 Department of Family Medicine, Kaohsiung Medical University Hospital, Kaohsiung Medical University, Kaohsiung, Taiwan; 4 Cmbio, Copenhagen, Denmark; 5 Department of Medical Sciences, Clinical Chemistry, Uppsala University, Uppsala, Sweden; 6 Department of Clinical Sciences in Malmö, Lund University, Malmö, Sweden; 7 School of Health and Social Studies, Dalarna University, Falun, Sweden

**Keywords:** atherosclerosis, gut microbiota, microbiome, salt, sodium, sodium chloride

## Abstract

**Introduction:**

High salt intake is associated with adverse health outcomes. Emerging evidence highlights the importance of the gut microbiome in human health, but large-scale human data on salt intake and the microbiome are limited. We examined the cross-sectional association between estimated 24-h sodium excretion (est24hNa) and the gut microbiome in a population-based cohort.

**Methods:**

We included 9,220 participants from the Swedish SCAPIS cohort with available shotgun metagenomic sequencing of fecal samples and urine analyses. We estimated the 24-h sodium excretion using the Kawasaki formula. Alpha diversity was assessed using the Shannon and inverse Simpson indices, and beta diversity using Bray–Curtis dissimilarity. Functional potential was evaluated using Gut Microbial Modules. Associations were analyzed using mixed linear regression models.

**Results:**

Alpha diversity was inversely associated with est24hNa, but associations were attenuated after adjustment for BMI and were not significant in fully adjusted models. Beta diversity was associated with est24hNa, although the explained variance was small (R2 < 0.001). Higher est24hNa was associated with 75 microbial species, including lower abundance of 27 species and higher abundance of oral–associated taxa such as *Streptococcus* spp. and *Veillonella*. It was also associated with increased abundance of pathways involved in microbial energy metabolism and carbohydrate fermentation.

**Conclusion:**

Higher est24hNa was associated with selected gut microbiome features, including a higher abundance of several oral-associated taxa and differences in inferred functional capacity for energy metabolism and carbohydrate fermentation. These findings identify microbial patterns associated with sodium exposure that may be relevant to cardiometabolic health.

## Introduction

Higher salt intake has well established adverse health effects (1). It raises blood pressure (2), alters the metabolic processes (3, 4) and promotes inflammation (5), thereby contributing to the atherosclerotic process. Reducing population-level intake is a priority for improving global health (6). Although the harmful effects of salt are extensively studied, the exact underlying pathological mechanisms are not fully understood.

The role of the gut microbiome in human health has emerged as an area of intense research interest. Interventional murine and rat studies have shown that salt intake can induce dysbiosis in bacterial composition (7–9), and disrupt the intestinal barrier (10, 11). These experimental findings suggest that such alterations may increase intestinal permeability and facilitate the translocation of microbial products, promoting systemic inflammation and vascular dysfunction, thereby providing a potential mechanistic link between salt consumption and increased cardiovascular disease risk. However, evidence from large human studies examining the association between sodium exposure and the gut microbiome composition remains limited. In the large, deeply phenotyped Swedish CArdioPulmonary bioImage Study (SCAPIS) cohort (12), we have previously demonstrated associations between salt intake, atherosclerosis (13), ventricular ectopy (14), and metabolic processes (3). In this context, we conducted an exploratory cross-sectional study of the relationship between estimated 24-h sodium excretion and gut microbiome composition, including alpha diversity, beta diversity, and species-level abundance, as well as microbial functional potential assessed using GMMs. The Bacillota/Bacteroidota ratio was examined as an additional analysis, as previous studies have reported associations between this ratio and salt intake (8–10).

## Materials and methods

### Study population

We used cross-sectional data from the SCAPIS cohort. SCAPIS was established to improve understanding of cardiovascular and pulmonary diseases through comprehensive imaging and extensive biomarker profiling; full cohort descriptions have been published previously (12, 13, 15). In brief, 59,909 randomly selected individuals aged 50–64 years from the general population in six Swedish regions were invited to participate, of whom 30,154 were included in the cohort (15). Participants underwent detailed phenotypic characterization, providing a rich dataset covering clinical, imaging, and biochemical measures.

For the present study, we included participants with available microbiota data and valid urinary sodium and urinary creatinine measurements from a single second-morning void urine from the Uppsala (*n* = 4,500) and Malmö (*n* = 4,806) study sites. Using the Kawasaki formula, we estimated 24-h sodium excretion (est24hNa) as a proxy for salt intake (16). Individuals with est24hNa values exceeding ±3 SD from the mean (*n* = 86) were excluded to reduce the influence of extreme outlying values on the analyses, resulting in a final analytical sample of 9,220 participants. One participant with an extremely low Shannon index was excluded for the analyses of alpha diversity (*n* = 9,219).

The Kawasaki formula:

est24hNa (g/day) = 1,000 × 22.99 × 16.3 x $\sqrt{X_{\mathrm{Na}.}}$ Where


$$X_{\text{Na}}=\frac{\mathrm{SM}U_{\mathrm{Na}}}{\mathrm{SM}U_{\mathrm{Cr}}}\;x\;(\text{PreCr}-\text{excretion})$$

SMU_Na_ (mmol/L)

SMU_Cr_ (mg/L)

Male:

PreCr-excretion (mg/day) = −12.63 x Age + 15.12 x Weight (kg) + 7.39 x Height (cm) – 79.9

Female:

PreCr-excretion (mg/day) = −4.72 x Age + 8.58 x Weight (kg) + 5.09 x Height (cm) – 74.5

### Microbiota data

Fecal samples were collected by participants at home, stored at −20 °C after delivery to the study center, shipped within 7 days to the central biobank, and stored at −80 °C until analysis.

DNA was extracted from fecal samples at Cmbio (Copenhagen, Denmark) using the NucleoSpin® 96 Soil kit (Macherey-Nagel). Genomic DNA was randomly fragmented to approximately 350 bp, and libraries were prepared using the NEBNext® DNA Library Prep Kit (New England Biolabs), followed by 2 × 150 bp paired-end sequencing on an Illumina platform.

Shotgun metagenomic reads were quality-filtered, and host-derived sequences were removed by alignment to the human reference genome GRCh38. The resulting non-host reads were taxonomically profiled using the CHAMP™ pipeline with the HMR05 reference database (17), with taxonomic annotations based on the Genome Taxonomy Database (GTDB) release r214; metagenome-assembled genomes without GTDB species-level annotations were clustered into species clusters using a ≥ 95% average nucleotide identity threshold.

### Gut metabolic modules (GMM)

Functional potential of the gut microbiota was assessed using gut metabolic modules (GMMs) (18), which are manually curated sets of functionally related genes representing defined microbial metabolic pathways and cellular processes.

### Statistical analysis

Descriptive statistics are presented as mean (standard deviation) for continuous variables and percentages for categorical variables.

Alpha diversity was assessed on down sampled relative abundances using the Shannon and inverse Simpson indices, which capture within-sample microbial diversity by accounting for both species richness and evenness. Based on previous analyses from the same cohort (13), we assumed linear associations between est24hNa and alpha diversity and analyzed these relationships using mixed linear models (lme4 package from R).

Bray–Curtis dissimilarity (calculated on down sampled relative abundances) was used to quantify beta diversity, and associations between est24hNa and the distance matrix were assessed using permutational multivariate analysis of variance (PERMANOVA) implemented in the adonis2() function from the vegan package in R.

We used the non-down sampled relative abundance data for the analysis and excluded rare taxas with a prevalence <5% or relative abundance < 10^−4^. The dataset was log-transformed to improve normality and analyzed using the Maaslin2 package. Statistical significance was defined as a False Discovery Rate (FDR)-adjusted *p*-value <0.05.

The functional potential of the gut microbiota was assessed using Gut Metabolic Modules (GMMs), analyzed in the same way as the taxonomic abundance data.

Three hierarchical models were specified to evaluate the robustness of the observed associations after adjustment of potential confounders/mediators. Model A was minimally adjusted for age and sex, with extraction plate included as a random effect to account for technical variation. Because extraction plates were site-specific, this also effectively adjusted for study site. Since BMI is strongly associated with both estimated sodium excretion and the gut microbiome, BMI was included separately in Model B to evaluate the extent to which it attenuated the observed associations. Finally, Model C additionally adjusted for established cardiovascular risk factors and dietary variables that could plausibly confound the relationship between estimated sodium excretion and the gut microbiome. All variables were added as fixed effects except from extraction plate that was added as random effect:

- Model A: Age, sex and extraction plate
- Model B: Model A + BMI (kg/m^2^)
- Model C: Model B +

  ° Systolic and diastolic blood pressure (measured as the average of two readings taken after a 5-min supine rest at inclusion),
  ° Diabetes mellitus (doctor-diagnosed or self-reported),
  ° Smoking status (current, former, or never smoker derived from questionnaire)
  ° Estimated glomerular filtration rate (eGFR, calculated using the revised Lund-Malmö formula (19))
  ° Total plasma cholesterol
  ° Total energy intake (calculated by a dietician from food frequency questionnaires - FFQs)
  ° Use of medications for hypertension, hyperlipidemia, diabetes mellitus (derived from questionnaires).
  ° Meat intake (processed and non-processed red meat and poultry from FFQs)
  ° Macronutrients (derived daily protein, fat and carbohydrate intake from FFQs)

Descriptive analysis of the model showed no indication of multicollinearity, as Variance Inflation Factors (VIF) were close to one for all covariates.

### Bacillota/Bacteroidota-ratio

An increased Bacillota/Bacteroidota-ratio (B/B-ratio - formerly Firmicutes/Bacteroidetes) has been associated with adverse health outcomes (20, 21) and has been commonly reported in rodent studies of high-salt diets (8–10). As a secondary targeted analysis, we therefore examined this ratio, calculated as the log-transformed relative abundance ratio with a pseudo count added to account for zeros. Associations with est24hNa were analyzed as a continuous exposure in Models A and C using mixed linear models. Outliers in the ratio were winsorized at 1st and 99th percentile to reduce the influence of extreme observations.

All statistical analyses were performed in R (version 4.5.1).

### Ethical statement

The SCAPIS-multicenter study was approved by the ethical review board at Umeå University, Sweden (number 2010–228-31 M) and the analysis of urine samples was approved by the ethical board at Uppsala University and Lund University, Sweden (number EPN Uppsala University 2016/387 and 2018/315; Lund University 2016/1031). All participants provided written informed consent for participation in SCAPIS.

## Results

Descriptive statistics of the participants are presented in Table 1 using the median est24hNa as cut-off between higher and lower. Participants with higher est24hNa had higher systolic and diastolic blood pressure, higher BMI, and a greater prevalence of diabetes mellitus. They were also more likely to use antihypertensive and/or lipid-lowering medications. Furthermore, individuals with higher est24hNa reported greater total energy, protein and carbohydrate intake as well as higher meat consumption.

**Table 1:** Descriptive statistics of included participants stratified by median est24hNa. Continuous variables are presented as mean (standard deviation) and categorical variables as percentages. Percentage of missing data is also reported.

	Lower est24hNa (*n* = 4,610)	Higher est24hNa (*n* = 4,610)	Missing data (%)
Est24hNa (g)	2.2 (0.6)	4.2 (0.9)	0
Systolic blood pressure (mmHg)	122 (16)	125 (16)	0.09
Diastolic blood pressure (mmHg)	75 (10)	77 (10)	0.11
Sex (% Women)	65	41	0
Age (years)	57 (4.4)	57 (4.3)	0
BMI, kg/m2	26 (4.3)	28 (4.5)	0
Smoking (%)			4.25
Current	13	12	
Never	52	54	
Former	34	34	
Diabetes Mellitus (%)	7.3	9.2	0.01
Cholesterol (mmol/l)	5.6 (1.1)	5.5 (1.1)	0.23
eGFR (ml/min/1.73 m^2^)	77 (10)	79 (10)	0.20
Hypertension medication (%)	18	20	4.59
Lipid medication (%)	7	8	4.56
Diabetes medication	3.2	4.2	4.57
Energy intake (kJ)	1,710 (697)	1,754 (743)	0
Red meat intake (g/day)	65 (43)	78 (50)	0
Poultry intake (g/day)	22 (18)	24 (19)	0
Fat intake (g/day)	69 (32)	70 (33)	0
Protein intake (g/day)	68 (26)	70 (27)	0
Carbohydrate intake (g/day)	182 (88)	189 (93)	0
Fiber (g/day)	20 (11)	20 (11)	0

### Alpha diversity

There was a significant negative association between est24hNa and both the Shannon and inverse Simpson indices in the minimally adjusted Model A (Shannon: *β* = −0.021, *p* < 0.001). Adding BMI as a covariate in Model B attenuated a large part of the association (*β* = −0.009, *p* = 0.018). No significant association remained in the fully adjusted Model C (Table 2, scatter plots with regression lines are presented in Supplementary Figures S1, S2).

**Table 2:** Statistics for the association between est24hNa, Shannon index and Inverse Simpson index (estimated 24 h Na-excretion in g).

Shannon index	*β*	95% CI	*p*-value
Model A	−0.021	−0.028 to −0.013	< 0.001
Model B	−0.009	−0.016 to −0.001	0.018
Model C	−0.004	−0.011 to 0.003	0.292
Inverse simpson			
Model A	−0.639	−0.879 to −0.399	<0.001
Model B	−0.305	−0.547 to −0.063	0.014
Model C	−0.164	−0.407 to 0.079	0.187

### Beta diversity

There was a significant association between est24hNa and Bray–Curtis dissimilarity in all models (PERMANOVA *p* < 0.001). Est24hNa explained only a small proportion of the variation in Bray–Curtis dissimilarity (*R*^2^ < 0.001 in all models).

### Abundance

Est24hNa was associated with 346, 109, and 75 species in regression Models A–C, respectively (Supplementary Tables). In the fully adjusted Model C, 27 species showed a negative association with est24hNa. The most statistically significant association was observed for *Latilactobacillus sakei*.

Among the associations remaining in the fully adjusted Model C, positively associated taxa included several members of oral-associated genera (e.g., *Streptococcus* spp., *Veillonella* spp.*, Actinomyces* spp.), many of which exhibit high fermentative capacity and contribute to carbohydrate and lactate metabolism.

Negatively associated taxa included anaerobic members of Lachnospiraceae, Ruminococcaceae, and Oscillospiraceae, including several putative short-chain fatty acid (SCFA) producers involved in complex carbohydrate fermentation.

**Figure 1:**
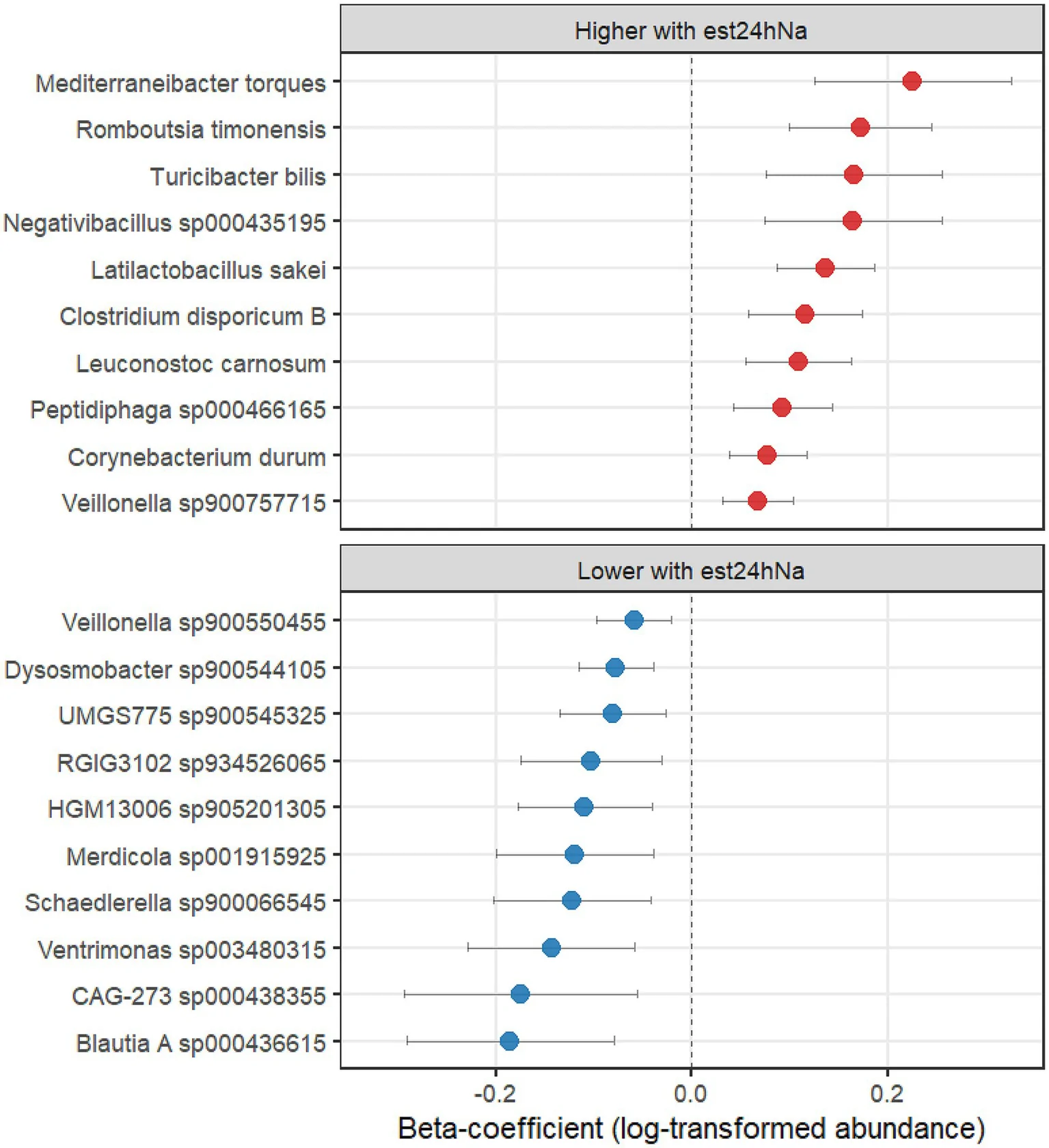
Top 10 significant positive and negative species associated with est24hNa (*q* < 0.05), with species ordered by *beta*-coefficient within each panel.

The top 10 positively and negatively associated species from Model C are shown in Figure 1, with spline curves presented in Supplementary Figure 3. Results for all significant findings are presented in Supplementary Tables Abundance model A–C.

### Gut metabolic modules

Est24hNa was associated with 51, 17 and 17 GMMs in regression model A to C. In the fully adjusted model C, only ethanol metabolism (ethanol production II – GMM ID: MF0091) had a negative association (Supplementary Tables GMM model A–C). The most statistically associated GMM was the non-oxidative branch pentose phosphate pathway (GMM ID: MF0071), classified under central energy metabolism (Table 3). Overall, the GMMs from model C are associated with carbohydrate metabolism (fructan, starch, lactose, galactose, fructose), SCFA-metabolism (acetate, lactate, propionate) and energy metabolism. All results for these models are presented in Supplementary Tables GMM model A-C.

**Table 3:** FDR-significant GMMs associated with est24hNa.

	Level 1	Level 2	GMM ID	Description	*β*	*p*_-FDR_
1.	central metabolism	energy metabolism	MF0071	pentose phosphate pathway (non-oxidative branch)	0.009	0.0009
2.	organic acid metabolism	propionate metabolism	MF0095	propionate production III	0.116	0.0009
3.	gas metabolism	hydrogen metabolism	MF0069	NADH:ferredoxin oxidoreductase	0.093	0.0014
4.	organic acid metabolism	acetate metabolism	MF0086	acetyl-CoA to acetate	0.009	0.0027
5.	central metabolism	energy metabolism	MF0073	pyruvate:ferredoxin oxidoreductase	0.010	0.0054
6.	central metabolism	energy metabolism	MF0067	glycolysis (preparatory phase)	0.006	0.0068
7.	alcohol metabolism	ethanol metabolism	MF0091	ethanol production II	−0.153	0.0164
8.	amino acid degradation	polar, uncharged amino acid degradation	MF0047	glutamine degradation II	0.011	0.0183
9.	organic acid metabolism	lactate metabolism	MF0092	lactate production	0.009	0.0243
10.	gas metabolism	hydrogen metabolism	MF0098	hydrogen metabolism	0.042	0.0249
11.	carbohydrate degradation	polysaccharide degradation	MF0002	fructan degradation	0.014	0.0268
12.	carbohydrate degradation	disaccharide degradation	MF0007	lactose and galactose degradation	0.102	0.0372
13.	carbohydrate degradation	polysaccharide degradation	MF0005	starch degradation	0.009	0.0382
14.	alcohol metabolism	ethanol metabolism	MF0090	ethanol production I	0.014	0.0453
15.	carbohydrate degradation	disaccharide degradation	MF0009	melibiose degradation	0.008	0.0481
16.	carbohydrate degradation	monosaccharide degradation	MF0017	galactose degradation	0.012	0.0485
17.	carbohydrate degradation	monosaccharide degradation	MF0015	fructose degradation	0.012	0.0499

### Bacillota/Bacteriodota-ratio

There was a positive, significant association between est24hNa and the B/B-ratio in model A (*β* = 0.023, *p* = 0.005). The significance was lost in the highly adjusted model C (β = 0.010, *p* = 0.243).

## Discussion

### Main findings

In this large population-based study, higher est24hNa was associated with differences in gut microbiota composition and inferred microbial functional potential.

We observed negative associations between est24hNa and alpha diversity (Shannon and inverse Simpson indices). These associations were substantially attenuated after adjustment for BMI and were no longer significant in fully adjusted models, suggesting that BMI, either as a confounder or potential mediator, may explain part of the observed relationship.

Est24hNa was also significantly associated with beta diversity, indicating overall differences in gut microbial community composition across the exposure gradient, although the proportion of explained variance was small.

In relative abundance analyses, 75 species were associated with est24hNa. Several positively associated taxa belonged to oral-associated genera, including Streptococcus and Veillonella, whereas other associated taxa, such as Latilactobacillus sakei and *Leuconostoc carnosum*, may reflect alternative mechanisms, including salt tolerance or food-related co-exposures. Negatively associated taxa included several anaerobic members of the Lachnospiraceae and Ruminococcaceae families.

Functional profiling identified a higher inferred functional potential for pathways related to carbohydrate utilization, central energy metabolism, and SCFA acid metabolism. Overall, these findings indicate coordinated taxonomic and inferred functional patterns associated with est24hNa, although the underlying biological mechanism are likely multifactorial.

### Comparisons with previous studies

Murine and rat studies have consistently shown that high salt intake alters gut microbiota composition (7–10), modulates SCFA production (7, 9), and promotes epithelial barrier disruption (10, 11, 22). Short-term high-salt challenges increase the Bacillota/Bacteroidota-ratio, deplete Lactobacillus species, and reshape the Ruminococcus- and Lachnospiraceae-related taxa communities. In the present study, we likewise observed a positive association between est24hNa and the Bacillota/Bacteroidota ratio, consistent with these experimental findings. However, this association was attenuated and no longer statistically significant after comprehensive adjustment, indicating that the initial association was partly explained by the included covariates.

The reshaping of the Ruminococcus- and Lachnospiraceae-associated taxa, together with alterations in SCFA–related pathways, is further concordant with our observations. In contrast, the depletion of Lactobacillus species reported in rodent models (7–10) and in one small short-term human intervention (23) study was not observed in our cohort. Lactobacillus did not emerge as a key taxon, suggesting that habitual salt intake in free-living populations may exert more subtle and adaptive compositional effects compared with acute experimental challenges.

Data on salt intake and the human gut microbiota are scarce. In addition to the small, lactobacillus-targeted study by Wilck et al. (23), Ferguson et al. (24) reported higher abundance of Bacillota and Ruminococcaceae in 135 healthy volunteers and identified Prevotella as a taxon associated with blood pressure. While our findings are consistent with enrichment of fermentative Bacillota-related taxa, Prevotella did not represent a dominant signal in our analysis.

To our knowledge, this is the first large-scale human study using shotgun metagenomics to comprehensively examine the relationship between est24hNa and the gut microbiome.

### Biological explanations

The intestinal barrier consists of epithelial cells covered by a mucus layer that supports a complex microbial ecosystem (25, 26). Microbial fermentation of carbohydrates generates SCFAs, particularly butyrate, which support epithelial integrity and mucus homeostasis (27, 28). Disruption of butyrate-producing bacteria has been linked to increased intestinal permeability, systemic inflammation, and cardiometabolic disease (27, 28).

In the present study, several microbiota alterations were consistent with potential perturbations in mucus-associated microbial ecology and barrier function, although alternative biological explanations may also account for some of the observed associations. *Akkermansia muciniphila*, a mucin-utilizing bacterium involved in balanced mucin turnover, was negatively associated with est24hNa (25). Loss of *A. muciniphila* has repeatedly been associated with impaired barrier function, as this species also supports the growth of butyrate-producing bacteria (29). Other negatively associated species belonged to the Lachnospiraceae and Ruminococcaceae families and included taxa involved in butyrate production and epithelial support (25, 29).

Conversely, mucin-degrading taxa including *Mediterraneibacter torques* and *Alistipes finegoldii* were positively associated with est24hNa and have previously been linked to states of mucus thinning and barrier dysfunction, although these associations are context-dependent (30, 31).

The observed pattern may also reflect ecological filtering. *A. muciniphila* is sensitive to elevated osmolality (32), whereas several oral-associated taxa such as *Streptococcus*, *Actinomyces*, and *Veillonella* spp. exhibit greater tolerance to environmental stress. The species with the strongest association, *Latilactobacillus sakei*, as well as *Leuconostoc carnosum*, are commonly found in fermented foods and are known to be salt tolerant, suggesting that salt tolerance or food-related co-exposures may contribute to some of the observed associations.

Gene module analysis identified a higher inferred functional potential for pathways related to carbohydrate fermentation, glycolysis, and the pentose phosphate pathway, as well as lactate production and downstream acetate and propionate metabolism. Several positively associated taxa participate in lactate-based cross-feeding networks typical of oral microbial communities, including lactate-producing streptococci and lactate-utilizing *Veillonella* species. These findings may reflect differences in the inferred functional potential of microbial fermentation pathways, although their implications for downstream SCFA production and gut metabolic ecology remain uncertain.

The presence of several oral-associated taxa in the gut microbiome, sometimes referred to as “oralization,” is of particular interest, as these organisms possess pro-inflammatory potential (33) and have been causally linked to atherosclerosis in experimental studies (34). In a prior study from the same cohort, oral-associated taxa were associated with prevalent atherosclerosis (35). Moreover, detection of streptococcal DNA in atherosclerotic plaques has been reported in multiple studies (36), suggesting biological plausibility for microbial involvement in plaque formation. While these observations provide biological context for the present findings, the potential link between sodium-associated microbial differences and vascular disease remains hypothetical.

Not all observed associations fit neatly within this framework. For example, *Faecalibacterium prausnitzii*, a prominent anaerobic butyrate-producing commensal, was positively associated with est24hNa, indicating that the observed pattern cannot be explained by a uniform loss of beneficial butyrate-producing bacteria. Overall, the findings likely reflect multiple, potentially overlapping biological processes, with alterations in mucus-associated microbial ecology representing one possible explanation.

### Strengths and limitations

This study has several important strengths. It is based on a large, well-characterized population-based cohort with extensive phenotypic data, including anthropometric, dietary, and biochemical measurements. The integration of these data with shotgun metagenomic sequencing of the fecal microbiome enables high-resolution, unbiased, and functionally informative characterization of host–microbiome relationships, representing a substantial advantage over targeted approaches such as 16S rRNA gene sequencing.

There are also limitations to consider. The cross-sectional design precludes causal inference and limits conclusions regarding directionality. Generalizability may be restricted, as the cohort consists exclusively of Swedish participants aged 50–64 years. In addition, information on medication use lacked sufficient specificity to fully account for potential drug-related effects on the microbiome. Fecal samples primarily reflect microbial communities in the distal colon, and because microbial composition varies along the gastrointestinal tract, the findings may not be representative of other intestinal regions. Because sodium intake is closely linked to habitual dietary patterns, adjustment for multiple dietary variables may partially account for foods that contribute substantially to sodium intake. While this reduces confounding by overall diet composition, it may also attenuate associations.

Sodium intake was estimated from a single spot urine sample using the Kawasaki equation, which was originally developed in a Japanese population (16) and has subsequently been validated against 24-h urine collections in other populations (37). Nevertheless, its application in a Swedish population may introduce population-specific estimation error. The equation has also been criticized for potentially generating spurious J-shaped associations due to correlations between its constituent variables and study outcomes (38, 39). In previous analyses of the same participants in the SCAPIS cohort, we observed positive linear associations between est24hNa and anthropometric measures, such as BMI, as well as blood pressure measures, rather than J-shaped relationships, consistent with theoretical expectations and findings based on 24-h urine measurements (3, 13, 40). Furthermore, simulation studies have shown that when sodium intake is held constant, the associations with outcomes are attenuated or disappear, suggesting that variability in sodium exposure, rather than the estimation method itself, drives the observed relationships (41).

Nevertheless, the limited precision of this method should be considered when interpreting absolute sodium values. The Kawasaki equation is not designed for individual-level assessment of sodium intake but is appropriate for ranking individuals within a population. Accordingly, the present findings should be interpreted within this epidemiological framework.

## Conclusion

In this large cross-sectional study based on a well-characterized Swedish cohort, high est24hNa was associated with selected taxonomic and functional features of the gut microbiome, including features of oralization and differences in mucus-associated species. Furthermore, higher est24hNa was associated with differences in the inferred functional potential for microbial energy metabolism and carbohydrate fermentation. Together, these findings identify microbial patterns associated with est24hNa and provide a basis for further studies investigating the potential role of the gut microbiome in the relationship between high sodium exposure and cardiovascular disease.

## Data Availability

The data analyzed in this study originate from SCAPIS and contain sensitive participant information and are subject to ethical, legal, and privacy restrictions. Requests to access the datasets should be directed to the corresponding author. Data access may be granted following application and approval according to the SCAPIS data access procedures.
