# Supplementary material for: Associations between sodium intake and gut microbiota composition
Source: Front Nutr. 2026 Sep 7;13:1889723. doi: 10.3389/fnut.2026.1889723 (PMC13592343; doi:10.3389/fnut.2026.1889723)

Supplementary figures

Supplementary figure 1: *Scatterplot with regression line showing the association between the Shannon index and estimated sodium intake (est24hNa)*

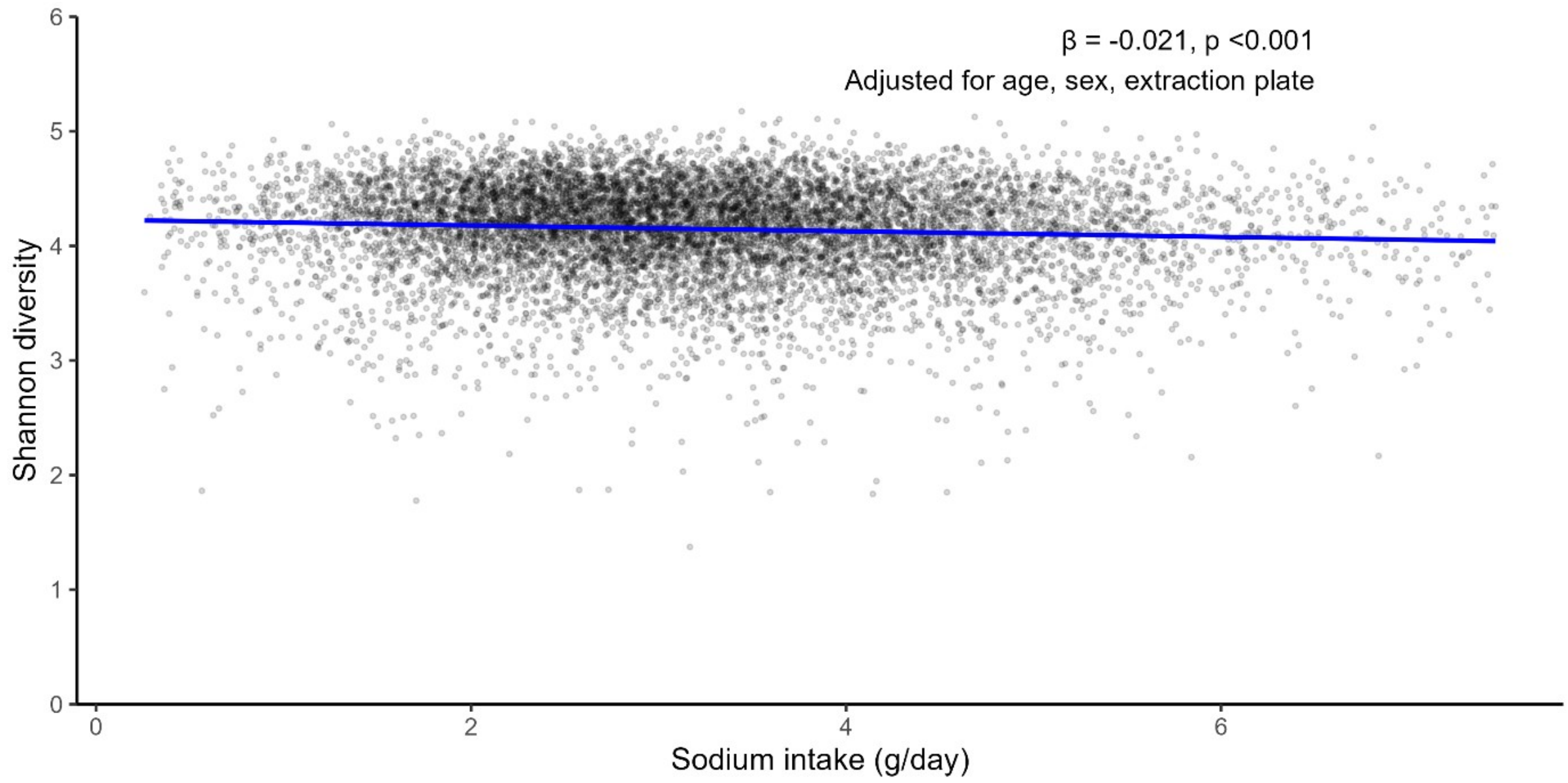

Supplementary figure 2: *Association between the Inverse Simpson index and estimated sodium intake (est24hNa). Scatterplot with regression line.*

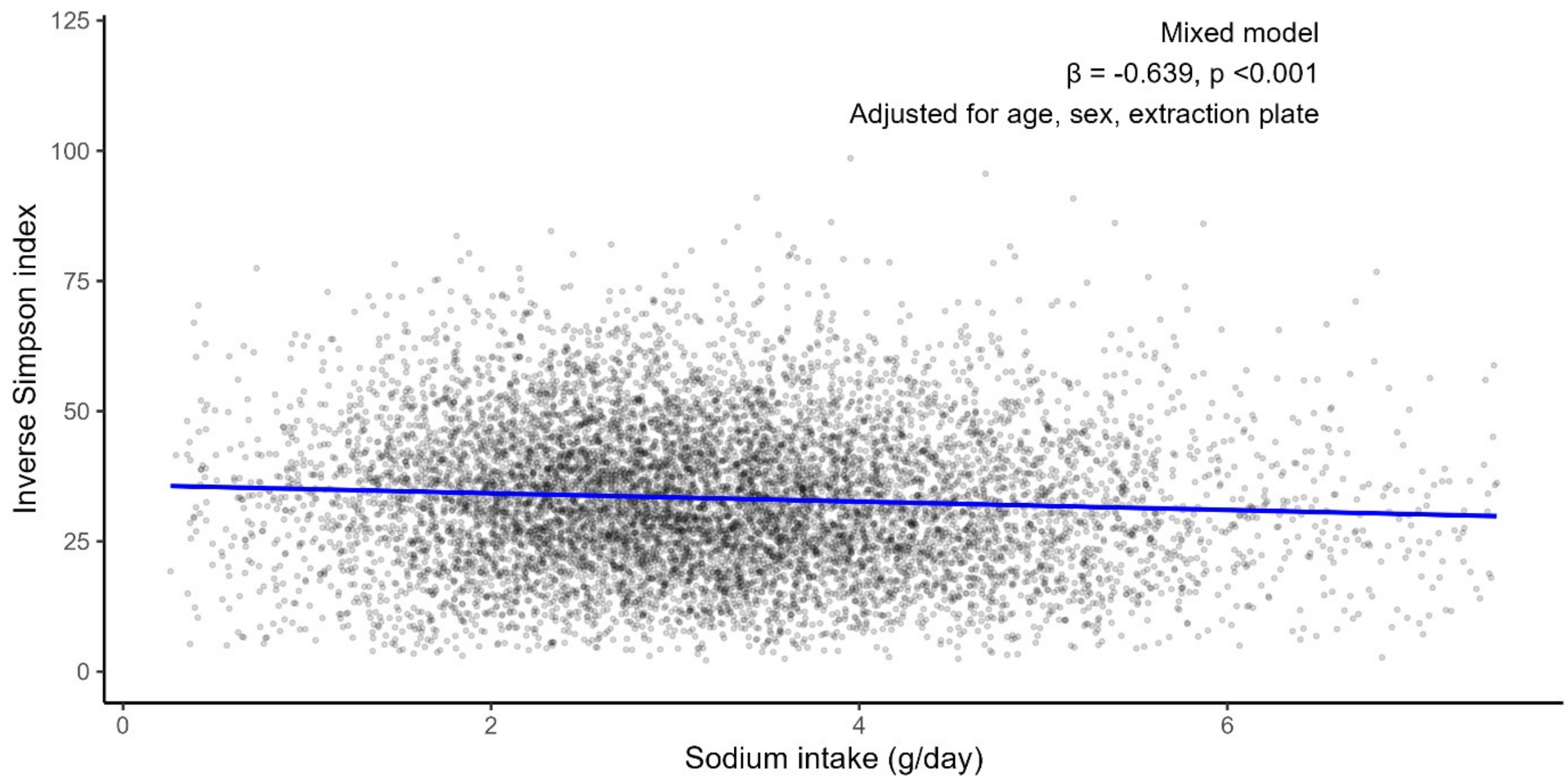

Supplementary figure 3: *Splines for the top ten associated taxas from Model C*

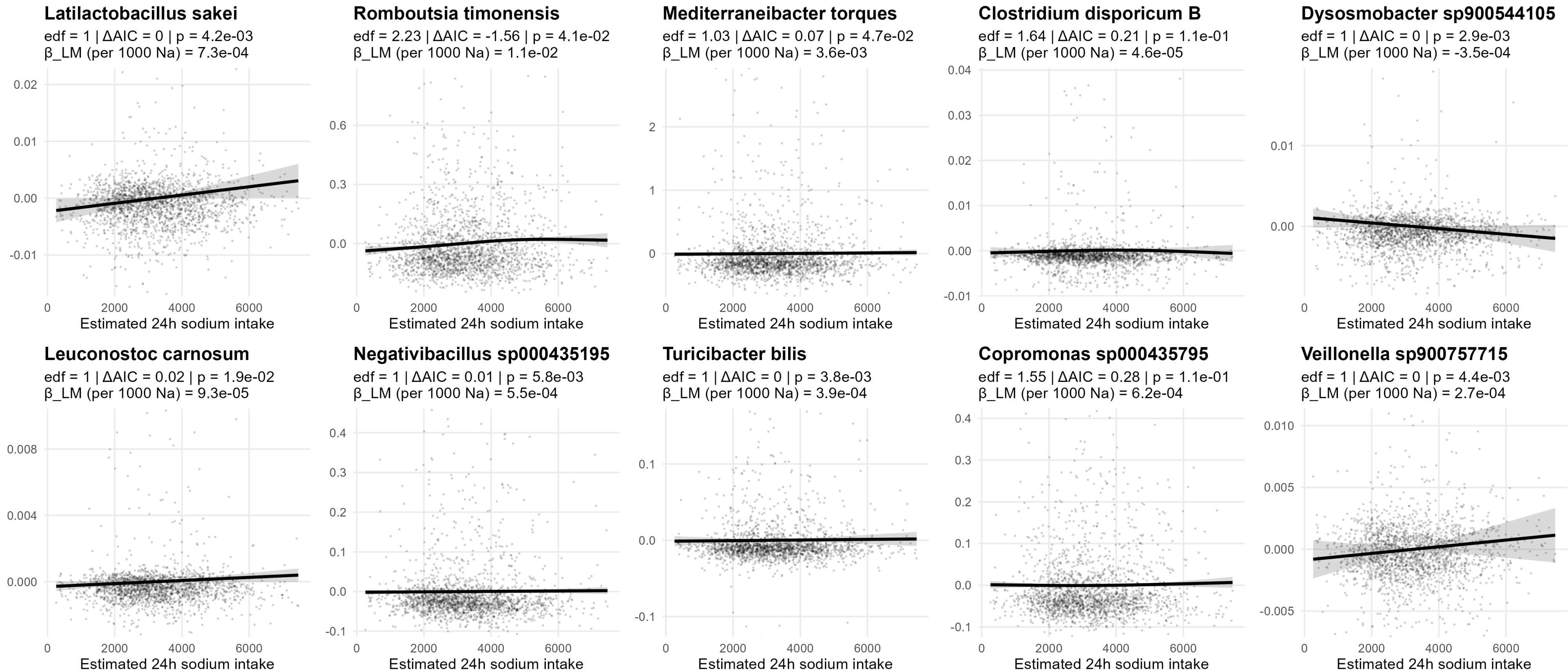

Supplement: Supplementary file 1 [file Data_Sheet_1.pdf]
